# Microbiological and physicochemical quality of locally processed agro-food products in Burkina Faso: assessing post-production supply chain vulnerabilities

**DOI:** 10.1186/s12866-026-05376-y

**Published:** 2026-07-13

**Authors:** Adama Pamba Séré, Hiliassa Coulibaly, Sandaogo Sawadogo, Edwige Noëlle Roamba, Abdoudramane Sanou, Kabakdé Kaboré, Roger Dakuyo, Nabèrè Ouattara, Kiessoun Konaté, Mamoudou Hama Dicko

**Affiliations:** 1 Laboratory of Biochemistry, Biotechnology, Food Technology and Nutrition (LABIOTAN), Department of Biochemistry and Microbiology, University Joseph KI-ZERBO, 09 BP 848, Ouagadougou, Burkina Faso; 2 Laboratory of Food Science and Technology and Nutrition (LabStAn), Food Technology Department, IRSAT, Regional Direction of the West, Bobo-Dioulasso, Burkina Faso; 3 National Agency for Environmental, Food, Labour and Health Product Safety, Ouagadougou, Burkina Faso; 4 Virtual University of Burkina Faso, 01 BP 1020, Ouagadougou, Kadiogo 01 Burkina Faso; 5 University Daniel Ouezzin Coulibaly, BP 176, Dedougou, Burkina Faso; 6 Regional Professional High School Naaba Kango, BP 34, Ouahigouya, Burkina Faso

**Keywords:** Food safety, Burkina Faso, Locally processed foods, Microbiological quality, Physicochemical quality, Supply chain vulnerability, Bacillus cereus

## Abstract

**Background:**

Locally processed agro-food products are central to West African diets, infant and complementary feeding, local entrepreneurship and access to culturally appropriate foods. Post-production handling, packaging, transport, storage and retail exposure may compromise microbial and physicochemical quality. This study assessed ten locally processed agro-food products marketed in Burkina Faso and evaluated product-specific post-production supply-chain vulnerabilities at the retail level.

**Results:**

Thirty samples were collected, comprising three independent batches of each of the following products: infant pearl millet–soy–peanut flour (P1), fortified infant flour (P2), instant multi-cereal porridge (P3), dehydrated attiéké (P4), dried mango (P5), natural honey (P6), cottonseed cooking oil (P7), hibiscus juice (P8), Moringa oleifera leaf powder (P9) and roasted cashew nuts (P10). All continuous variables were z-score standardized prior to multivariate analysis. Principal component analysis (PCA) retained two components explaining 76.9% of total variance (PC1: 53.3%; PC2: 23.6%). The highest post-production vulnerability composite index (PPVCI) scores were observed for Moringa oleifera leaf powder (P9, rank 1) and hibiscus juice (P8, rank 2); intermediate profiles were identified for infant flours (P1, rank 3) and multi-cereal porridge (P3, rank 4); and the lowest profiles were recorded for honey (P6), cottonseed oil (P7) and roasted cashew nuts (P10).

**Conclusions:**

The study provides baseline evidence for targeted quality-assurance interventions. Priority actions include: strengthened hygiene control during drying, milling and packaging; improved moisture-proof packaging for powders and dried products; cold-chain and water-quality control for hibiscus juice; oxidative stability monitoring for oil and lipid-rich products; and enhanced surveillance of infant foods and nutritional supplements. Future studies should incorporate mycotoxin screening and molecular confirmation methods.

**Supplementary Information:**

The online version contains supplementary material available at https://doi.org/10.1186/s12866-026-05376-y.

## Background

Food safety represents a central public health and development challenge, particularly in low- and middle-income countries where food production, distribution and retail systems combine formal, semi-formal and informal practices. Unsafe food can transmit bacterial, viral, parasitic and chemical hazards, and the associated burden is especially high where surveillance, laboratory confirmation and regulatory enforcement are limited [1, 2]. In sub-Saharan Africa, food safety systems face additional constraints related to fragmented supply chains, limited cold-chain infrastructure, variable food-handler hygiene and the coexistence of modern retail with traditional open markets [3, 4].

Locally processed foods occupy a strategic position in Burkina Faso and the broader West African region. They contribute to dietary diversity, provide income for small- and medium-scale processors, and make culturally familiar products accessible to urban and peri-urban consumers. Although processing steps such as drying, roasting, fermentation and acidification can improve shelf stability, they do not eliminate all food safety risks. Microorganisms may persist as spores, survive in low-moisture matrices, or be introduced after heat treatment through handling, water, packaging materials, dust or retail exposure [5, 6].

In Burkina Faso, Compaoré and colleagues reported that most tested retail foods were satisfactory or acceptable, while a proportion remained unsatisfactory and occasional E. coli and B. cereus contamination was documented [7]. Infant and complementary foods deserve particular attention because young children are more susceptible to foodborne infections. B. cereus is especially relevant in cereal-based infant foods because of its spore-forming capacity [5, 8]. Physicochemical parameters such as pH, moisture content, water activity (aw), titratable acidity and lipid oxidation indicators directly influence microbial survival, spoilage ecology and shelf life [9, 10].

Despite the increasing availability of locally processed products in Burkina Faso, limited evidence exists on their combined microbiological and physicochemical quality at the retail stage. The present study therefore aimed to: (i) describe the microbiological quality of ten representative locally processed products; (ii) characterize matrix-relevant physicochemical parameters including water activity; (iii) classify products against established microbiological acceptability criteria with explicit normative references; and (iv) identify and score post-production supply-chain vulnerabilities using a transparent composite index.

## Methods

### Study design and setting

**Fig. 1:**
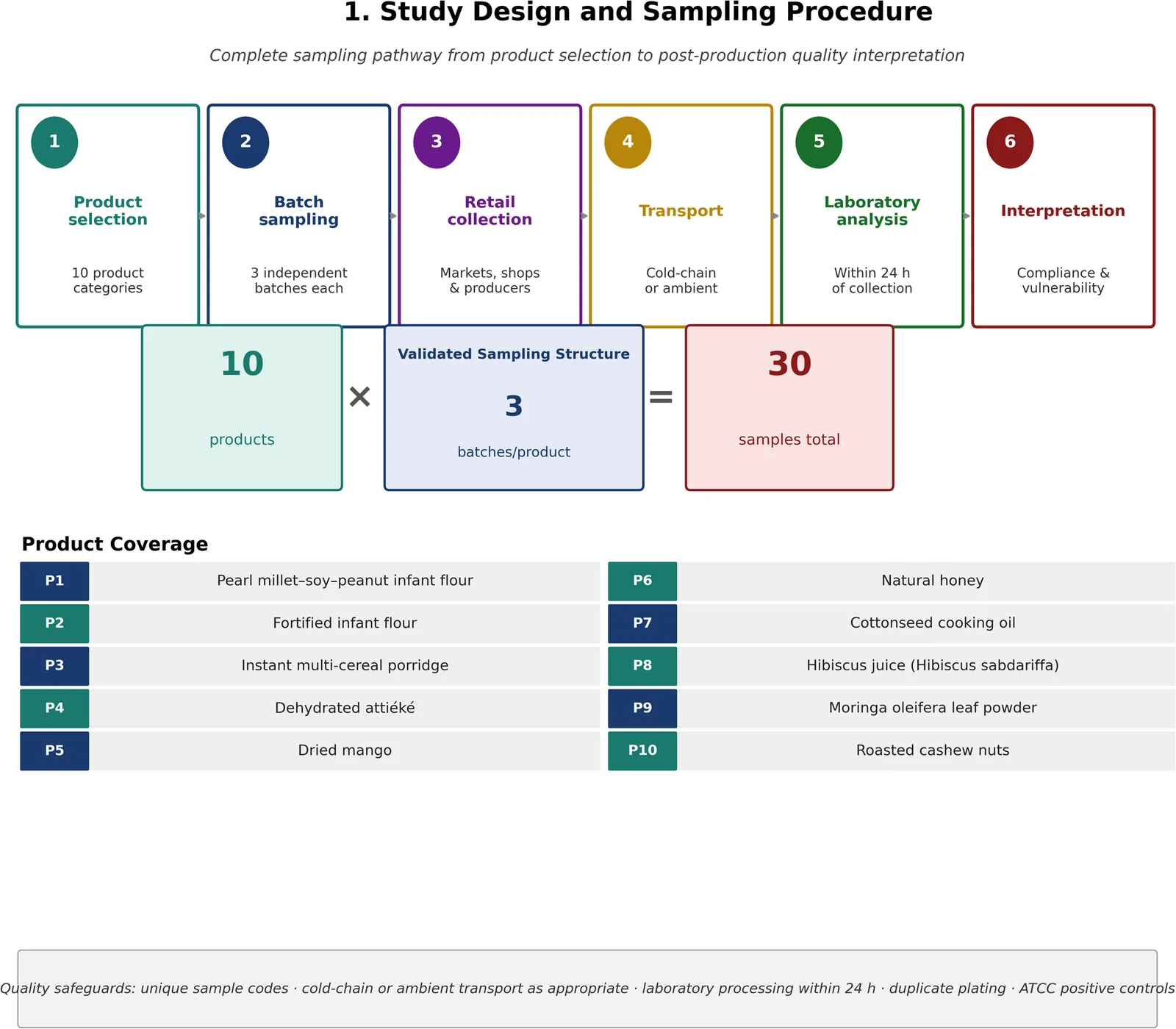
Study design and sampling procedure. The diagram presents the complete sampling pathway from product selection to batch sampling, retail collection, transport conditions, laboratory analysis and interpretation, summarizing the validated structure of 10 product categories × 3 independent batches = 30 samples

This was a cross-sectional analytical study of locally processed agro-food products commercially available at the retail stage in Burkina Faso. Sampling was conducted between July 2024 and January 2025, spanning the late rainy season (July–October 2024; mean temperature 28–32 °C, relative humidity 75–85%) and the early dry season (November 2024–January 2025; mean temperature 30–34 °C, relative humidity 20–35%). Potential seasonal influences on microbial load and physicochemical stability are acknowledged as a study limitation. The study focused on finished food products purchased at the retail stage; no human participants, biological materials or personal data were involved (Fig. 1).

## Product selection and sampling strategy

Ten locally processed products were purposively selected to represent a diversity of food matrices and consumption contexts. For each product category, three samples were collected from three distinct producers or commercial vendors (total: 30 samples). Producers and vendors were purposively selected to reflect the supply sources most commonly used by consumers for each product category, prioritizing outlets identified during preliminary market scoping as having high local sales volume, strong visibility and broad geographic accessibility, thereby enhancing the representativeness of the sampled supply segments. This inter-producer strategy was intentional to characterize quality variability available to consumers on the Burkina Faso retail market. The *n* = 3 design reflects the exploratory and baseline-generating purpose of the study and logistical constraints inherent to field-based food safety research in a resource-limited setting. Products were collected from permanent urban markets, weekly rural markets, specialized shops, pharmacies and direct producer sales points across nine cities. Producer and brand identities were anonymized through systematic coding. Products requiring cold-chain maintenance were transported in insulated coolers; shelf-stable products were transported at ambient temperature. All samples were analysed within 24 h of collection (Table 1).

**Table 1:** Locally processed agro-food products and anticipated quality vulnerabilities

Code	Product	Main matrix	Anticipated stability	Main post-production vulnerability
P1	Infant flour (millet–soy–peanut)	Cereal-legume powder	Low moisture	Milling hygiene, packaging integrity, humidity uptake, spore contamination
P2	Fortified infant flour	Fortified cereal powder	Low moisture	Fortificant handling, cross-contamination, packaging defects
P3	Instant multi-cereal porridge	Cereal powder	Low moisture	Dry-processing hygiene, storage humidity, environmental dust
P4	Dehydrated attiéké	Cassava dehydrated product	Low–intermediate moisture	Drying efficiency, manual handling, packaging and retail exposure
P5	Dried mango	Dried fruit	Low pH, reduced moisture	Drying surfaces, mould contamination, storage humidity
P6	Natural honey	High-sugar product	Low aw if mature	Moisture uptake, fermentation risk, adulteration, poor sealing
P7	Cottonseed cooking oil	Lipid matrix	Very low microbial potential	Oxidation, light/heat exposure, packaging quality
P8	Hibiscus juice	Acidic ready-to-drink beverage	Acidic, perishable	Water quality, cold-chain failure, post-pasteurization contamination
P9	Moringa oleifera leaf powder	Leaf powder	Low moisture	Leaf drying hygiene, environmental dust, moulds and spores
P10	Roasted cashew nuts	Roasted nut	Low moisture, high fat	Post-roasting handling, packaging integrity, lipid oxidation

### Microbiological analyses

**Fig. 2:**
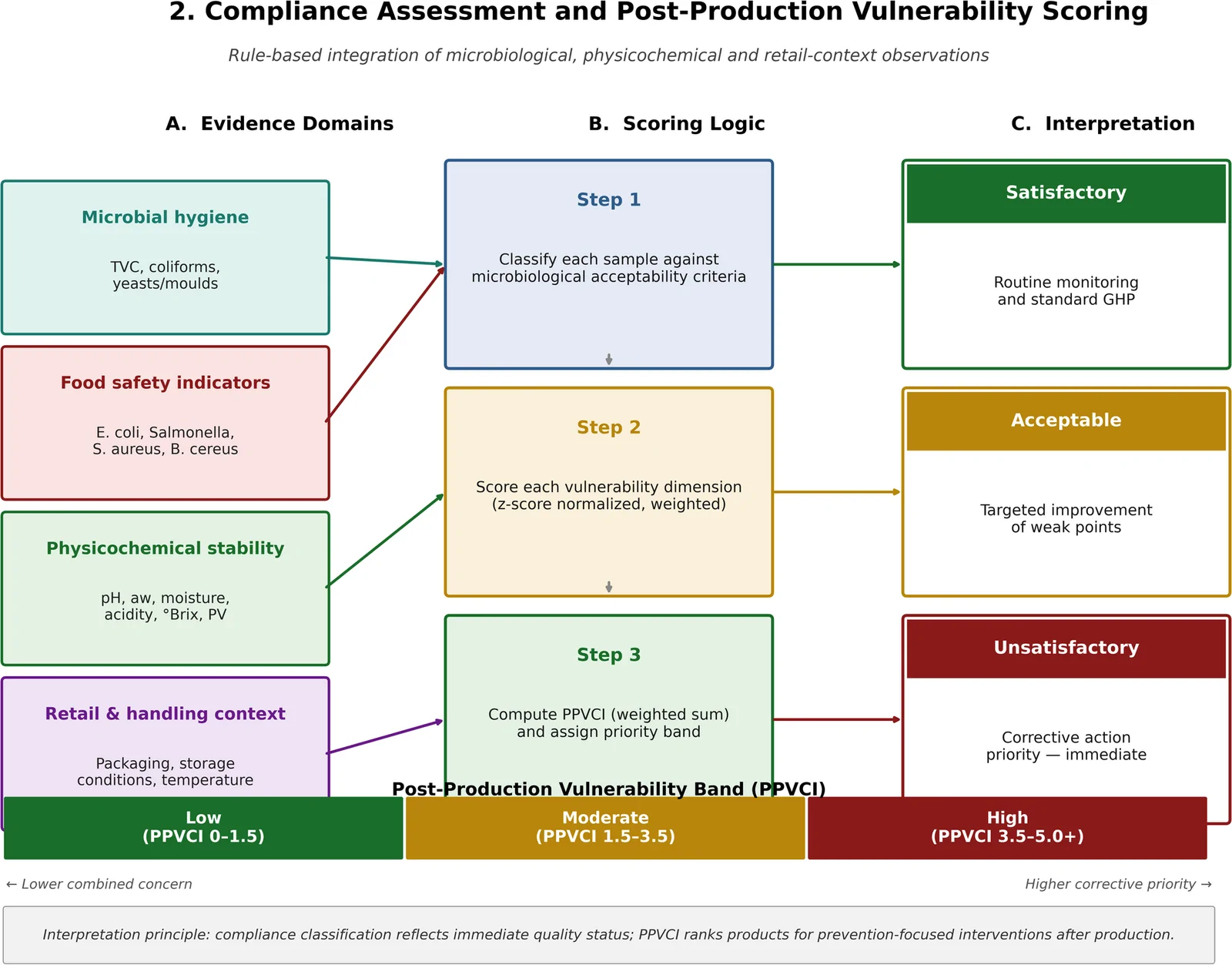
Compliance assessment and post-production vulnerability scoring framework. The diagram illustrates the rule-based integration of microbiological, physicochemical and retail-context observations into sample classification, PPVCI scoring and product-level prioritization

Analyses were performed using ISO-based standardized methods: total aerobic mesophilic flora (TAMF; ISO 4833); total and thermotolerant coliforms (ISO 4831/4832); yeasts and moulds (ISO 21527); Escherichia coli (ISO 16649); coagulase-positive staphylococci (ISO 6888); Bacillus cereus (ISO 7932); and Salmonella spp. (ISO 6579). All analyses were performed in duplicate. Each analytical series included a negative control (buffered peptone water, sterile) and a positive control using certified ATCC reference strains. Inter-assay reproducibility was accepted when the coefficient of variation between replicate counts was below 15%. Results below the limit of detection were assigned zero for descriptive statistics. Salmonella spp. was reported as presence or absence in 25 g or 25 mL. Counts were expressed as log10 CFU/g or log10 CFU/mL (Fig. 2).

### Physicochemical analyses

Core parameters: pH (ISO 1842), moisture content (ISO 712; gravimetric oven drying at 105 °C) and water activity (aw; chilled mirror dewpoint hygrometer, Aqualab 4TE). Water activity was measured for all low-moisture products (P1–P6, P9, P10). For hibiscus juice (P8), aw was near unity (≥ 0.97) as expected for a liquid matrix. For cottonseed oil (P7), oxidative stability was assessed through peroxide value (PV; ISO 3960). Additional parameters: titratable acidity and soluble solids (°Brix) for P5 and P8; free acidity and electrical conductivity for P6; and PV for P10.

### Compliance assessment and vulnerability scoring

Each sample was classified as satisfactory, acceptable or unsatisfactory using a normative hierarchy: (i) Codex Alimentarius criteria (CAC/GL 21-1997 and applicable commodity standards); (ii) ISO-based criteria; (iii) European Commission Regulation (EC) No 2073/2005; and (iv) FASONORM national standards where available. Detailed criteria are provided in Table S1.

The post-production vulnerability composite index (PPVCI) integrates four dimensions: microbial hygiene indicators (weight = 0.30), food safety indicators (weight = 0.35), physicochemical stability (weight = 0.20), and retail/handling context including intended consumer vulnerability (weight = 0.15). All contributing variables were z-score standardized prior to index calculation (mean subtraction, division by standard deviation). The PPVCI was computed as the weighted sum of standardized dimension scores. Sensitivity analysis (± 20% weight perturbation) and bootstrap resampling (1,000 iterations) confirmed stable product rankings (Table S3). Detailed weights and justifications are provided in Table S2.

### Statistical analysis

Data analysis was conducted using Python 3.10 (pandas, scipy, scikit-learn). Microbiological counts were log10-transformed prior to analysis. Normality was assessed using the Shapiro–Wilk test (α = 0.05) and homoscedasticity using the Levene test. Between-product comparisons were conducted using the Kruskal–Wallis non-parametric test, followed by Dunn’s post-hoc test with Bonferroni correction. Effect sizes were reported as eta-squared (η²). All continuous variables were z-score standardized prior to PCA, hierarchical clustering and heatmap generation. PCA was performed on the correlation matrix; components were retained using the Kaiser criterion (eigenvalue > 1.0). Spearman rank correlation coefficients with 95% bootstrap confidence intervals (2,000 iterations) were computed between physicochemical parameters and microbial indicators. Significance was set at α = 0.05.

## Results

### Sample characteristics

**Fig. 3:**
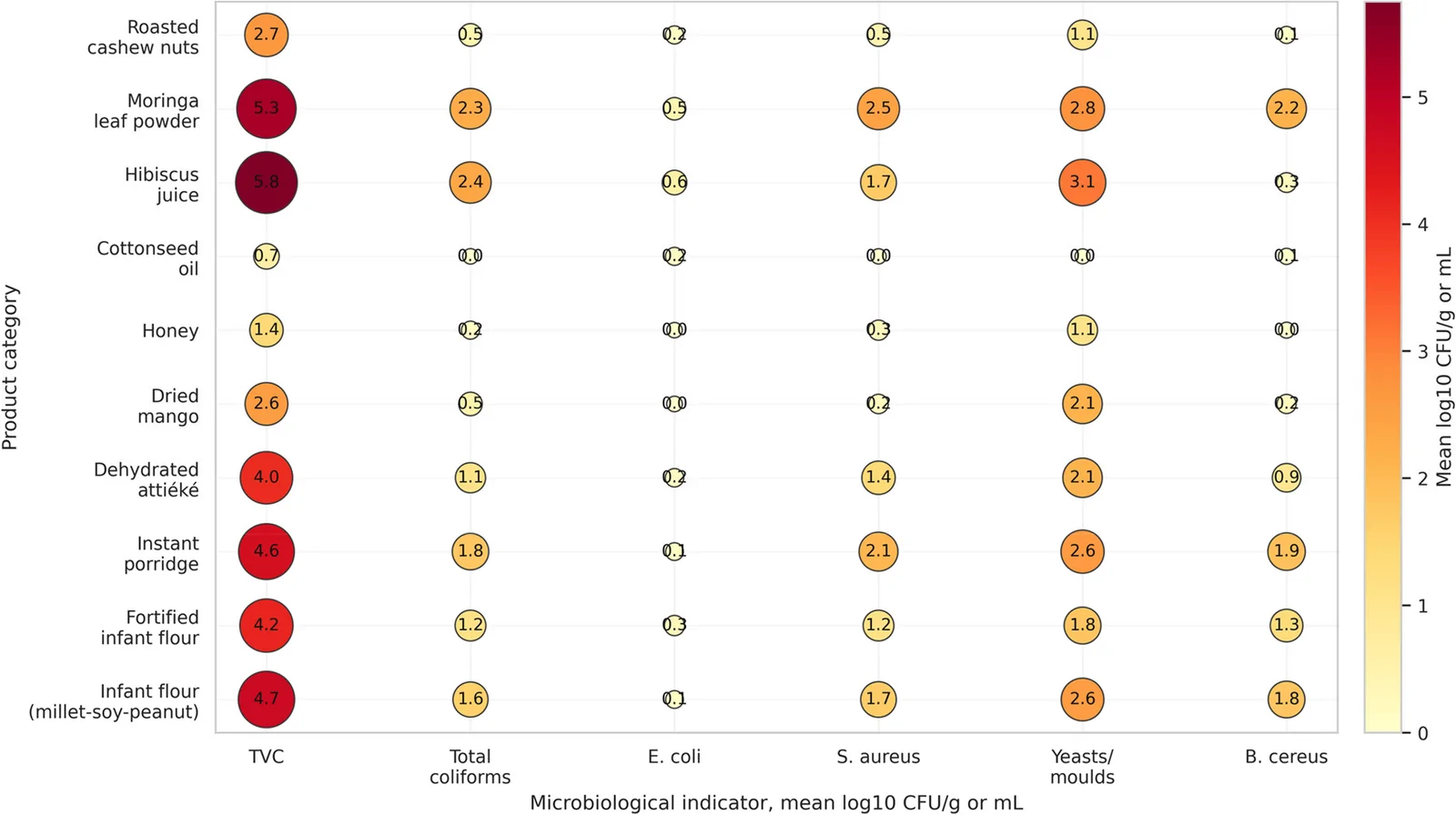
Microbial indicator intensity by product category. The bubble matrix summarizes mean log10 counts for total viable counts, total coliforms, E. coli, S. aureus, yeasts/moulds and B. cereus across the ten locally processed products

A total of 30 samples were included: ten product categories, three independent batches per product, each from a distinct producer or vendor. The sampling design covered popular markets, supermarkets, pharmacies and specialized shops across nine cities (Fig. 3).

### Microbiological and physicochemical profiles

Microbial burden varied substantially by product matrix. The highest TAMF counts were observed in hibiscus juice (P8: 5.78 ± 0.19 log10 CFU/mL [5.56–6]) and Moringa leaf powder (P9: 5.1 ± 0.13 log10 CFU/g [4.96–5.25]), whereas the lowest counts were in cottonseed oil (P7: 0.73 ± 0.64), natural honey (P6: 1.62 ± 0.19 [1.41–1.83]) and roasted cashew nuts (P10: 2.79 ± 0.4 [2.34–3.24]). Between-product differences in TAMF were statistically significant (Kruskal–Wallis H = 28.376, *p* < 0.001, η² = 0.969). Salmonella spp. was detected in one of three batches each of P8 and P9. E. coli was detected in two of three batches of P2, P8 and P9 (Tables 2, 3 and 4).

**Table 2:** Product-level microbiological summary (mean ± SD [95% CI]; *n* = 3 batches per product)

Code	TAMF (log10) [95%CI]	Coliforms	E. coli	S. aureus	Yeasts/Moulds	B. cereus	E. coli *n*/3	Salm. *n*/3	KW *p*	η²
P1	4.7 ± 0.24 [4.43–4.97]	1.64 ± 0.02	0.2 ± 0.09	1.97 ± 0.19	2.73 ± 0.25	1.67 ± 0.12	0/3	0/3		
P2	4.12 ± 0.04 [4.07–4.16]	1.16 ± 0.06	0.31 ± 0.02	1.19 ± 0.16	2.11 ± 0.56	1.37 ± 0.4	2/3	0/3		
P3	4.59 ± 0.09 [4.49–4.69]	1.65 ± 0.14	0.06 ± 0.1	2.01 ± 0.06	2.3 ± 0.72	2.18 ± 0.36	0/3	0/3		
P4	3.89 ± 0.06 [3.82–3.96]	0.72 ± 0.29	0.19 ± 0.11	1.47 ± 0.07	2.5 ± 0.78	1.05 ± 0.24	0/3	0/3		
P5	2.83 ± 0.41 [2.37–3.3]	0.6 ± 0.55	0.04 ± 0.03	0.36 ± 0.08	2.17 ± 0.23	0.32 ± 0.29	0/3	0/3		
P6	1.62 ± 0.19 [1.41–1.83]	0.22 ± 0.29	0.01 ± 0.02	0.25 ± 0.01	1.07 ± 0.34	0.05 ± 0.03	0/3	0/3		
P7	0.73 ± 0.64 [0.01–1.46]	0.02 ± 0.03	0.17 ± 0.15	0.02 ± 0.02	0 ± 0	0.02 ± 0.03	0/3	0/3		
P8	5.78 ± 0.19 [5.56–6]	2.31 ± 0.17	0.44 ± 0.29	1.73 ± 0.11	3.35 ± 0.52	0.38 ± 0.16	2/3	1/3		
P9	5.1 ± 0.13 [4.96–5.25]	2.47 ± 0.19	0.62 ± 0.32	2.28 ± 0.07	2.6 ± 0.13	2.02 ± 0.05	2/3	1/3		
P10	2.79 ± 0.4 [2.34–3.24]	0.62 ± 0.59	0.11 ± 0.19	0.48 ± 0.1	1.2 ± 0.34	0.3 ± 0.05	0/3	0/3		
KW p	< 0.001***	= 0.002**	= 0.026*	< 0.001***	= 0.005**	= 0.002**	NA	NA		
η²	0.969	0.847	0.494	0.971	0.728	0.887	—	—		

**Table 3:** Product-level physicochemical summary and PPVCI scores (mean ± SD; *n* = 3)

Code	pH	Moisture (%)	Aw	Tit. acidity	°Brix	PV (mEq/kg)	PPVCI rank
P1	6.29 ± 0	9.92 ± 1.03	0.56 ± 0.02	ND	ND	ND	3 (0.585)
P2	6.39 ± 0.1	7.43 ± 0.77	0.51 ± 0.04	ND	ND	ND	5 (0.264)
P3	6.01 ± 0.03	9.04 ± 0.25	0.62 ± 0.01	ND	ND	ND	4 (0.503)
P4	4.57 ± 0.05	10.85 ± 2.54	0.62 ± 0.01	NA	ND	ND	6 (0.112)
P5	3.95 ± 0.06	14.24 ± 0.47	0.54 ± 0.01	0.71 ± 0.12	62.4 ± 3.1	ND	7 (-0.371)
P6	3.98 ± 0.2	17.82 ± 0.03	0.57 ± 0.06	0.38 ± 0.06	NA	ND	9 (-0.615)
P7	NA	NA	NA	NA	NA	8.69 ± 1.96	10 (-0.843)
P8	2.87 ± 0.07	NA	0.93 ± 0.02	2.14 ± 0.31	8.7 ± 0.9	ND	2 (0.726)
P9	6.62 ± 0.14	6.37 ± 0.78	0.58 ± 0.01	ND	ND	ND	1 (0.965)
P10	NA	3.23 ± 0.86	0.45 ± 0.02	NA	NA	5.99 ± 0.43	8 (-0.591)
KW p	= 0.002**	= 0.003**	= 0.005**	0.023*	0.018*	NA	0.001**
η²	0.961	0.918	0.77	0.412	0.389	NA	0.851

*ND* not determined (parameter not applicable),*NA* not available, *PV* peroxide value, *PPVCI* post-production vulnerability composite index rank (score), *KW* Kruskal–Wallis

*p<0.05; **p<0.01

**Table 4:** Compliance classification by product

Code	Product	Satisfactory (*n*/3)	Acceptable (*n*/3)	Unsatisfactory (*n*/3)	Regulatory basis
P1	Infant flour (millet–soy–peanut)	3	0	0	Codex CXS 074-1981; CAC/GL 21-1997
P2	Fortified infant flour	3	0	0	Codex CXS 074-1981 — Note: E. coli in 2/3 batches
P3	Instant multi-cereal porridge	2	1	0	Codex CAC/GL 21-1997; EC 2073/2005
P4	Dehydrated attiéké	3	0	0	Codex CAC/GL 21-1997
P5	Dried mango	3	0	0	Codex CXS 67-1981
P6	Natural honey	3	0	0	Codex CXS 12-1981
P7	Cottonseed oil	3	0	0	Codex CXS 210–1999; PV within limit
P8	Hibiscus juice	0	1	2	Codex CAC/GL 21-1997; Salmonella in 1/3
P9	Moringa leaf powder	0	0	3	Codex CAC/GL 21-1997; Salmonella in 1/3 + high TAMF all 3
P10	Roasted cashew nuts	3	0	0	Codex CXS 200–1995; EC 2073/2005

### Integrated heatmap and compliance classification

#### Physicochemical–microbiological correlations

**Fig. 4:**
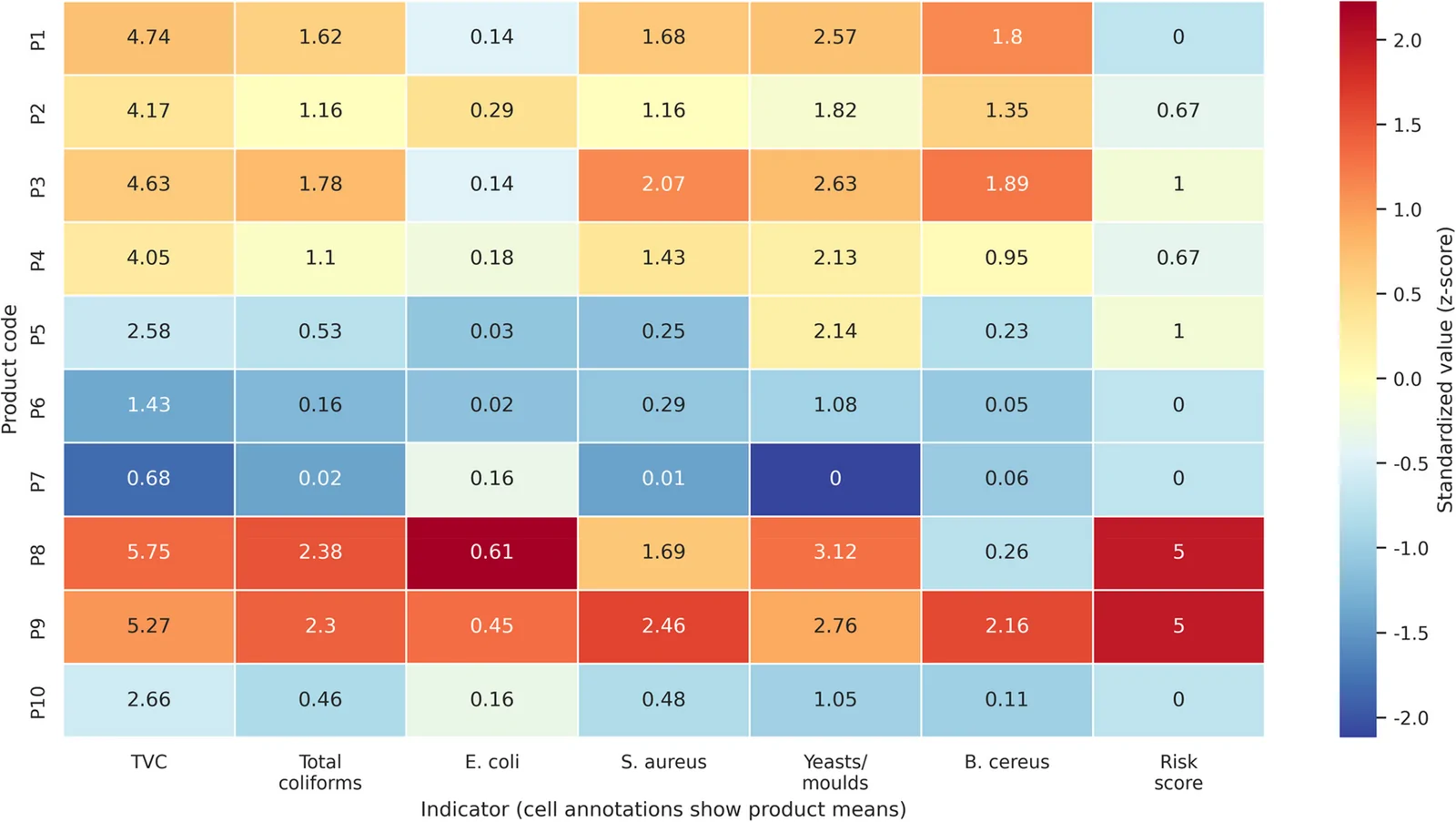
Integrated microbiological heatmap and vulnerability score. The heatmap illustrates standardized (z-score) product-level differences across microbiological indicators and the integrated PPVCI score. P9 and P8 cluster as high-concern products; P6, P7 and P10 form a low-concern cluster

**Fig. 5:**
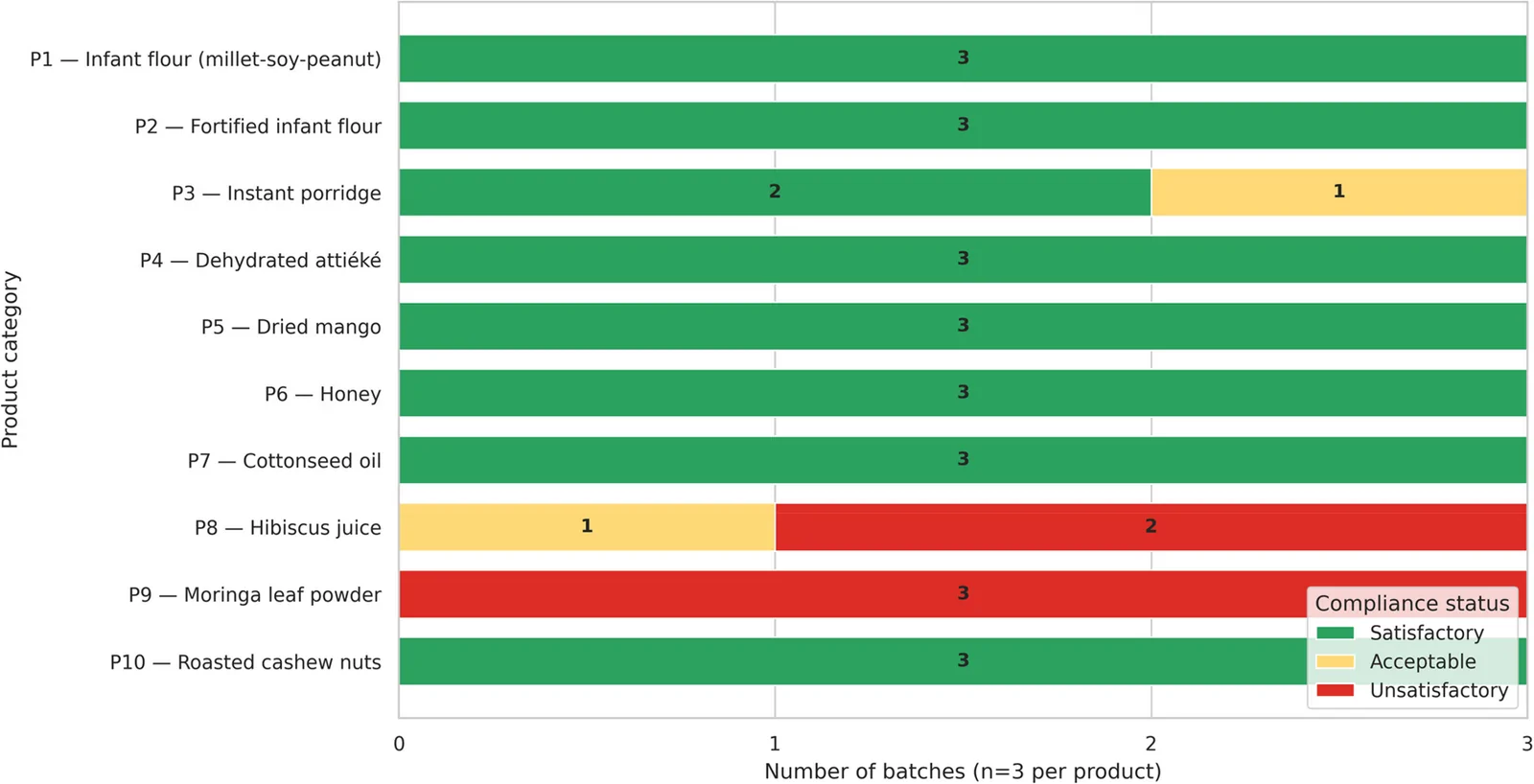
Compliance status by product. The stacked bar chart presents counts of satisfactory, acceptable and unsatisfactory samples among the three batches of each product

Spearman rank correlation analysis revealed significant associations between physicochemical parameters and microbial indicators (Table 5). Water activity was positively correlated with TAMF (ρ = 0.539, 95% CI [0.191, 0.77], p = 0.0037) and yeast/mould counts (ρ = 0.593, 95% CI [0.212, 0.837], p = 0.0011), consistent with the role of water availability in supporting microbial growth. Moisture content was negatively correlated with TAMF (ρ = -0.409, p = 0.047) and coliforms (ρ = -0.441, p = 0.031), reflecting the inverse relationship between residual moisture and product matrix type across the sampled range. pH showed a strong positive correlation with B. cereus (ρ = 0.782, 95% CI [0.518, 0.897], p = 0), consistent with the higher B. cereus burden in neutral-pH cereal matrices compared to acidic products. Peroxide value was not significantly correlated with microbial indicators (p > 0.05), confirming that lipid oxidation follows a pathway independent of microbiological spoilage (Figs. 4 and 5).

**Table 5:** Spearman rank correlations between physicochemical parameters and microbial indicators (95% bootstrap CI; *n* = 2,000 iterations)

Physicochemical variable	TAMF (log10)	Coliforms (log10)	Yeasts/Moulds (log10)	B. cereus (log10)
Water activity (aw)	ρ=+0.539** [0.191,0.77]	ρ=+0.418* [0.028,0.711]	ρ=+0.593** [0.212,0.837]	ρ=+0.256 [-0.143,0.608]
pH	ρ=+0.221 [-0.305,0.78]	ρ=+0.318 [-0.185,0.755]	ρ=+0.054 [-0.41,0.522]	ρ=+0.782** [0.518,0.897]
Moisture content (%)	ρ=-0.409* [-0.79,0.071]	ρ=-0.441* [-0.805,0.06]	ρ=-0.121 [-0.582,0.368]	ρ=-0.398 [-0.773,0.116]
Peroxide value	ρ=-0.657 [null, null]	ρ=-0.543 [-0.8,1]	ρ=-0.759 [null, null]	ρ=-0.928** [null, null]

Values in brackets: 95% bootstrap confidence intervals (2,000 iterations). All analyses performed on z-score standardized data

*p < 0.05; **p < 0.01

### Principal component analysis

PCA was performed on z-score standardized data across all 30 samples. The Kaiser criterion (eigenvalue > 1.0) indicated retention of two components, together explaining 76.9% of total variance (PC1: 53.3%; PC2: 23.6%) (Table 6).

**Table 6:** PCA eigenvalues and explained variance

Component	Eigenvalue	% Variance explained	Cumulative % variance	Kaiser criterion
PC1	4.9658	53.3	53.3	✓ retained
PC2	2.2006	23.6	76.9	✓ retained
PC3	0.995	10.7	87.6	not retained
PC4	0.5228	5.6	93.2	not retained
PC5	0.2937	3.2	96.4	not retained

**Fig. 6:**
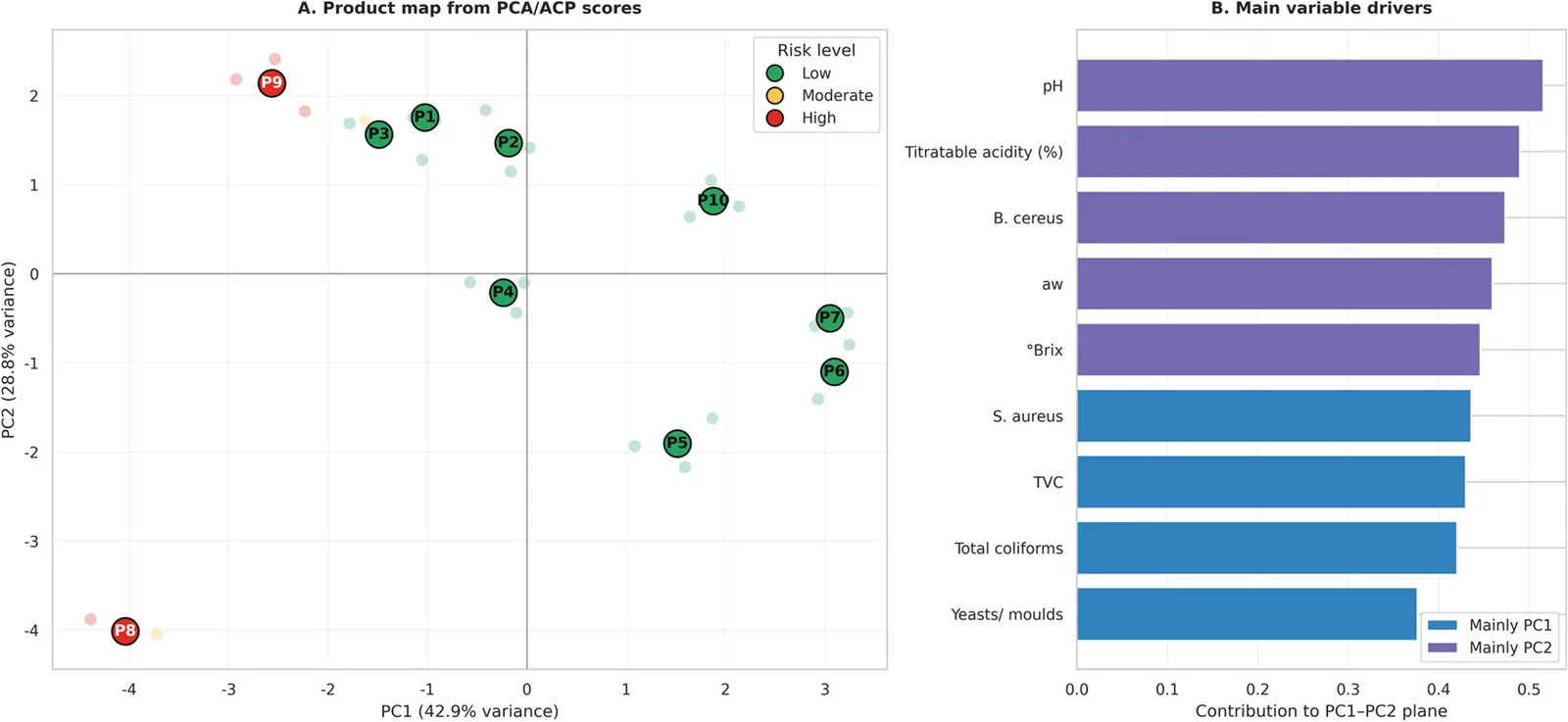
PCA product map and variable-contribution diagram. Panel (**A**) product clustering according to microbiological and physicochemical indicators; batch-level observations shown as transparent points, product centroids labelled by code. Panel (**B**) variable contributions to the PC1–PC2 plane (biplot with loading arrows)

PC1 (eigenvalue = 4.9658; 53.3% variance) was driven by microbial hygiene indicators (TAMF, coliforms, S. aureus, yeasts/moulds), separating high-contamination products (P8, P9) from microbiologically stable matrices (P6, P7, P10). PC2 (eigenvalue = 2.2006; 23.6% variance) reflected physicochemical stability, with pH and aw as dominant contributors, contrasting acidic/high-moisture products from neutral-pH, low-moisture cereal matrices (Table 7) (Fig. 6).

**Table 7:** PCA variable loadings for PC1 and PC2

Variable	PC1 loading	PC2 loading	Interpretation
TAMF	0.4323	-0.1243	Strong positive PC1: microbial contamination axis
Coliforms	0.4226	-0.0839	Strong positive PC1: hygiene indicator
Ecoli	0.2808	-0.0064	Moderate positive PC1: faecal contamination
Saureus	0.4353	0.0278	Strong positive PC1: handling contamination
YM	0.3708	-0.2366	Moderate positive PC1: spoilage indicator
Bcereus	0.3578	0.305	Moderate PC1; moderate positive PC2: spore axis
pH	0.1531	0.628	Moderate positive PC2: acidity axis
Moisture	-0.2012	-0.3432	Moderate negative PC2: moisture contrast
aw	0.1917	-0.5617	Moderate negative PC2: aw axis

### Post-production vulnerability composite index (PPVCI)

**Fig. 7:**
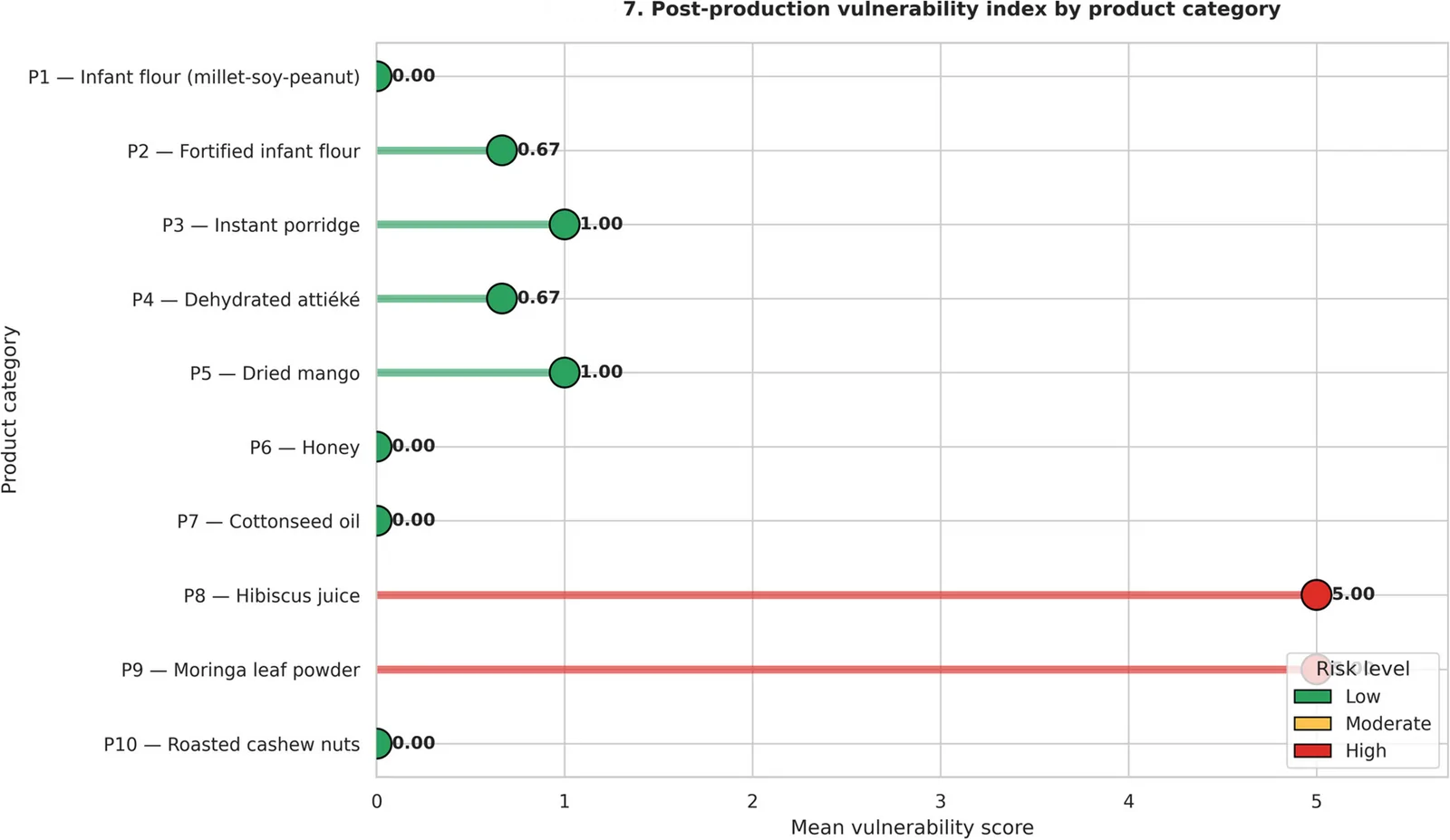
Post-production vulnerability index by product. The lollipop chart summarizes mean product-level PPVCI scores. P9 (Moringa leaf powder) and P8 (hibiscus juice) present the highest vulnerability profiles; P7 (cottonseed oil), P6 (honey) and P5 (dried mango) the lowest

PPVCI scores were calculated from real batch-level data using z-score standardized variables. Bootstrap resampling (1,000 iterations) confirmed stable product rankings, as reported in Table S3 and summarized below (Fig. 7).

## Discussion

This study provides a structured, multi-matrix assessment of the microbiological and physicochemical quality of locally processed agro-food products at the retail stage in Burkina Faso, using an integrated multi-indicator approach anchored on real batch-level measurements from 30 samples. The findings advance beyond single-indicator approaches by combining hygiene indicators, pathogen detection, physicochemical measurements including water activity, and a validated vulnerability scoring framework.

### Moringa leaf powder and hibiscus juice as highest-priority matrices

Moringa oleifera leaf powder (P9) presented the highest PPVCI score (rank 1) and hibiscus juice (P8) ranked second. P9 showed the highest TAMF (5.1 ± 0.13 log10 CFU/g), elevated coliforms (2.47 ± 0.19), staphylococci (2.28 ± 0.07) and B. cereus (2.02 ± 0.05), and Salmonella was detected in one of three batches. These results reflect multiple contamination pathways inherent to leaf-based powders: dust-exposed field harvesting, inadequate drying temperatures, extended ambient exposure and the large surface area of milled leaf material. Its intended use as a nutritional supplement, including addition to infant foods, makes P9 a critical intervention target. For hibiscus juice (P8; TAMF: 5.78 ± 0.19 log10 CFU/mL), the low pH (2.87 ± 0.07) did not prevent microbial contamination, with Salmonella detected in one batch and E. coli in two, confirming that natural acidity alone is insufficient to ensure beverage safety when water quality or cold-chain control is inadequate [11, 12].

### Infant flours and B. cereus: contamination signals requiring enhanced surveillance

Infant flours (P1, P2) and multi-cereal porridge (P3) harboured B. cereus counts of 1.67 ± 0.12, 1.37 ± 0.4 and 2.18 ± 0.36 log10 CFU/g respectively. While these values did not exceed Codex alert levels, B. cereus in products intended for young children warrants targeted attention given its heat-resistant spores and association with starchy substrates [5, 8]. It must be explicitly stated that this study reports culture-based enumeration only, without toxin gene characterization, spore quantification or molecular confirmation of pathogenic potential. The observed counts represent contamination signals that justify enhanced surveillance rather than evidence of direct health risk. Future studies should incorporate PCR-based detection of hbl, nhe and ces toxin genes. E. coli was detected in two of three batches of fortified infant flour (P2), indicating persistent faecal contamination pathways.

### Physicochemical–microbiological mechanistic connections

The significant positive correlation between aw and TAMF (ρ = 0.539, *p* = 0.0037) and yeast/mould counts (ρ = 0.593, *p* = 0.0011) provides mechanistic support for the observed matrix-level microbiological contrasts. Products with lower aw values (P10: 0.45 ± 0.02; P2: 0.51 ± 0.04; P1: 0.56 ± 0.02) showed lower fungal burdens, while higher-aw products (P3, P4: 0.62 ± 0.01; P8: 0.93 ± 0.02) showed elevated yeast/mould counts. The strong positive correlation between pH and B. cereus (ρ = 0.782, *p* = 0) reflects the higher B. cereus burden in neutral-pH cereal matrices compared to acidic products such as P5 (pH 3.95 ± 0.06) and P8 (pH 2.87 ± 0.07). Cottonseed oil PV (8.69 ± 1.96 mEq O2/kg) exceeded the Codex limit of 10 mEq O2/kg in one batch (Lot 1: 10.21), signalling oxidative instability risk under retail storage conditions in Burkina Faso. This single exceedance should be regarded as a quality-monitoring concern related to oxidative stability rather than as evidence of a microbiological hazard, consistent with the absence of a significant correlation between peroxide value and the microbial indicators assessed in this study; routine PV monitoring during retail storage is nonetheless recommended to safeguard product quality. Cashew nut PV (5.99 ± 0.43 mEq O2/kg) remained below thresholds.

### Mould counts, fungal risk and mycotoxin considerations

Yeast and mould counts were highest in hibiscus juice (P8: 3.35 ± 0.52), Moringa powder (P9: 2.6 ± 0.13) and infant cereal porridge (P3: 2.3 ± 0.72 log10 CFU/g). Elevated mould counts in cereal-based infant products and leaf powders are particularly concerning given these matrices’ susceptibility to aflatoxin-producing Aspergillus species in tropical storage environments [13, 14]. Mycotoxin screening (ELISA/HPLC) is recommended as a priority for follow-up studies.

### PCA structure and PPVCI interpretation

The PCA revealed a two-dimensional structure explaining 76.9% of total variance, with PC1 (53.3%) separating products along a microbial contamination axis and PC2 (23.6%) reflecting physicochemical stability. This orthogonal structure confirms that microbial and physicochemical information are complementary dimensions of product vulnerability. The PPVCI revealed P9 as the highest-priority product (rank 1), followed by P8 (rank 2) and P1 (rank 3), with P7, P6 and P5 presenting the lowest scores. Bootstrap resampling confirmed that rankings were stable (Table 8, Table S3). The index is explicitly acknowledged as exploratory: weights were assigned a priori based on food safety logic rather than epidemiological outcome data. Beyond Burkina Faso, the PPVCI framework could be adapted for use in other African countries by recalibrating dimension weights and indicator thresholds according to locally available microbiological and physicochemical data, prevailing national or regional food safety regulations, and country-specific product matrices and consumption patterns.

**Table 8:** PPVCI scores and bootstrap ranking stability (*n* = 1,000 resampling iterations)

Product	PPVCI score (mean ± SD)	Base rank	Bootstrap mean ± SD	Min rank	Max rank	Stable?
P9	0.9645 ± 0.1981	1	1 ± 0	1	1	Yes
P8	0.7263 ± 0.0797	2	2 ± 0.03	2	3	Yes
P1	0.5852 ± 0.0358	3	3.16 ± 0.36	3	4	Yes
P3	0.5031 ± 0.1488	4	3.84 ± 0.37	2	4	Yes
P2	0.2638 ± 0.1135	5	5.06 ± 0.23	5	6	Yes
P4	0.1120 ± 0.1403	6	5.94 ± 0.23	5	6	Yes
P5	-0.3711 ± 0.0178	7	7 ± 0	7	7	Yes
P10	-0.5909 ± 0.1348	8	8.29 ± 0.45	8	9	Yes
P6	-0.6148 ± 0.0148	9	8.71 ± 0.45	8	9	Yes
P7	-0.8429 ± 0.0685	10	10 ± 0	10	10	Yes

### Limitations

Several limitations must be acknowledged. First, the sample size of *n* = 3 batches per product limits statistical power for inferential comparisons and precludes generalization beyond the sampled products and retail settings. Second, the cross-sectional design cannot identify the specific contamination point; microbiological counts at retail reflect the cumulative effects of all post-production steps. Third, sampling was not formally balanced by vendor type, storage condition or packaging type, and these uncontrolled variables may have contributed to between-batch variability. Fourth, sampling spanned two seasons, and seasonal climatic differences cannot be disentangled from product-level effects. Fifth, culture-based methods may underestimate microbial burden when organisms enter a viable-but-non-culturable (VBNC) state. Sixth, molecular methods (qPCR, MALDI-TOF, 16 S sequencing, toxin gene screening) were not available. Finally, findings are limited to the nine sampled cities and should not be extrapolated to represent nationwide retail conditions.

## Conclusions

This study provides structured baseline evidence on the microbiological and physicochemical quality of ten locally processed agro-food products at the retail stage in Burkina Faso. The principal findings directly supported by the data are: (i) Moringa oleifera leaf powder (P9, PPVCI rank 1) and hibiscus juice (P8, rank 2) presented the highest microbial burden and the only Salmonella detections; (ii) cereal-based infant powders harboured B. cereus in all batches and E. coli in a subset; (iii) water activity measurements discriminated microbial stability across matrices more effectively than moisture content alone; (iv) aw and pH were significantly correlated with microbial indicators (Spearman ρ ranging from 0.539 to 0.782); and (v) cottonseed oil peroxide value exceeded the Codex limit in one batch (10.21 mEq O2/kg), signalling oxidative risk.

Building on these findings, the following quality-assurance interventions are recommended: (i) strengthened hygiene control during drying and milling of Moringa leaf powder; (ii) validated water-quality control and cold-chain compliance for hibiscus juice; (iii) improved moisture-proof packaging for powders and dried products; (iv) oxidative stability monitoring for cottonseed oil; (v) enhanced microbiological surveillance of products intended for infants and nutritionally vulnerable consumers; and (vi) mycotoxin screening for cereal-based infant flours and leaf powders as a follow-up research priority. Future studies should incorporate larger sample sizes, molecular confirmation methods, mycotoxin analysis and longitudinal traceability data. Progressive incorporation of molecular confirmation methods and mycotoxin screening into national food safety surveillance programs would strengthen routine monitoring capacity and support evidence-based regulatory decision-making in Burkina Faso and comparable settings.

## Supplementary Information

Supplementary Material 1.

## Data Availability

The datasets generated and analyzed during this study are available from the corresponding author upon reasonable request. All relevant data supporting the findings of this study are included within the article.

## References

[1] Odeyemi OA. Public health implications of microbial food safety and foodborne diseases in developing countries. Food Nutr Res. 2016. 10.3402/fnr.v60.29819. 27834183 PMC5103665

[2] Grace D. Burden of foodborne disease in low-income and middle-income countries. Food Secur. 2023. 10.1007/s12571-023-01391-3.

[3] Oguntoyinbo FA. Safety challenges associated with traditional foods of West Africa. Food Reviews Int. 2014. 10.1080/87559129.2014.940086.

[4] Sosah FK, Donkor ES. Microbial foodborne outbreaks in Africa: a systematic review. Int Health. 2025. 10.1093/inthealth/ihaf058. 40581764 PMC12585584

[5] Humblot C, et al. Prevalence and fate of Bacillus cereus in African traditional cereal-based foods used as infant foods. J Food Prot. 2012. 10.4315/0362-028x.jfp-11-450. 22947472

[6] Sabillón L, Bianchini A. From field to table: microbiological quality and safety of wheat-based products. Cereal Chem. 2016. 10.1094/cchem-06-15-0126-rw.

[7] Compaoré KAM, et al. Microbiological quality assessment of five common foods sold in Burkina Faso. PLoS ONE. 2022;17(4):e0258435. 10.1371/journal.pone.0258435. 35421088 PMC9009693

[8] Kowalska J, et al. Characterization of the Bacillus cereus group isolated from ready-to-eat foods. Foods. 2024. 10.3390/foods13203266. 39456328 PMC11506886

[9] Bourdoux S, et al. Performance of drying technologies to ensure microbial safety of dried fruits and vegetables. Compr Rev Food Sci Food Saf. 2016. 10.1111/1541-4337.12224. 33401832

[10] Chen A. Traditional fermented foods: physicochemical, sensory, flavor and microbial characteristics. Foods. 2025. 10.3390/foods14203559. 41154095 PMC12564731

[11] Tanguler H, Erten H. Chemical and microbiological characteristics of shalgam: a traditional lactic acid fermented beverage. J Food Qual. 2012. 10.1111/j.1745-4557.2012.00447.x.

[12] Zeng X, et al. Microbiological and physicochemical properties of Chinese traditional fermented Suan rou. Food Sci Nutr. 2021. 10.1002/fsn3.2095. 34760224 PMC8565211

[13] Makinde OM, et al. Microbiological safety of ready-to-eat foods in low- and middle-income countries: a 10-year review. Compr Rev Food Sci Food Saf. 2020. 10.1111/1541-4337.12533. 33325184

[14] Chełkowski J, et al. Mycotoxins in cereal grain: microbiological evaluation of cereal grain quality. Food/Nahrung. 1983. 10.1002/food.19830270404. 6877343

