# Supplementary material for: Microbiological and physicochemical quality of locally processed agro-food products in Burkina Faso: assessing post-production supply chain vulnerabilities
Source: BMC Microbiol. 2026 Jul 13;26:797. doi: 10.1186/s12866-026-05376-y (PMC13560229; doi:10.1186/s12866-026-05376-y)
Supplement: Supplementary file 1 [file 12866_2026_5376_MOESM1_ESM.docx]

**Supplementary Material**

**Table S1. Microbiological acceptability criteria by product and normative reference.**

| **Product** | **Criterion** | **Threshold** | **Normative reference** | **Note** |
| --- | --- | --- | --- | --- |
| P1, P2 — Infant flours | TAMF | ≤ 5×10⁴ CFU/g | Codex CXS 074-1981; CAC/GL 21-1997 | Infant formula standard |
| P1, P2 — Infant flours | Salmonella spp. | Absent in 25 g | Codex CXS 074-1981 | Zero tolerance |
| P1, P2 — Infant flours | B. cereus | ≤ 10² CFU/g (alert) | ISO 7932; expert guidance | Spore-former; starchy matrices |
| P3 — Multi-cereal porridge | TAMF | ≤ 10⁵ CFU/g | CAC/GL 21-1997; EC 2073/2005 | Cereal-based product |
| P4 — Dehydrated attiéké | TAMF | ≤ 10⁵ CFU/g | CAC/GL 21-1997 | Cassava-based dried product |
| P5 — Dried mango | Yeasts/moulds | ≤ 10³ CFU/g | Codex CXS 67-1981 | Dried fruit standard |
| P6 — Honey | Moisture | ≤ 20% | Codex CXS 12-1981 | Fermentation risk above 20% |
| P6 — Honey | aw | ≤ 0.60 | Codex CXS 12-1981 | Osmophilic yeast growth risk |
| P7 — Cottonseed oil | Peroxide value | ≤ 10 mEq O2/kg | Codex CXS 210-1999 | Refined vegetable oil |
| P8 — Hibiscus juice | Salmonella spp. | Absent in 25 mL | CAC/GL 21-1997 | Ready-to-drink; zero tolerance |
| P9 — Moringa powder | TAMF | ≤ 10⁵ CFU/g | CAC/GL 21-1997 | Leaf powder supplement |
| P10 — Roasted cashew nuts | TAMF | ≤ 10⁴ CFU/g | Codex CXS 200-1995 | Tree nut standard |
| All products | E. coli | Absent or < 10 CFU/g | CAC/GL 21-1997; EC 2073/2005 | Faecal indicator |

**Table S2. PPVCI dimension variables, weights and justification.**

| **Dimension** | **Variables included** | **Weight** | **Justification** |
| --- | --- | --- | --- |
| Microbial hygiene | TAMF, total coliforms, yeasts/moulds | 0.30 | Overall process hygiene and storage conditions (FAO/WHO framework) |
| Food safety indicators | E. coli, Salmonella, coag.-positive staphylococci | 0.35 | Direct public health hazard — highest weight |
| Physicochemical stability | pH, aw, moisture, titratable acidity, PV (where applicable) | 0.20 | Indirect risk modifiers; influence microbial survival and spoilage |
| Retail/handling context + consumer vulnerability | Packaging integrity, storage conditions, consumer group (infant/adult) | 0.15 | Contextual exposure amplifiers — qualitative but essential |

**Table S3. PPVCI sensitivity analysis and bootstrap ranking stability.**

| **Product** | **Base PPVCI** | **Base rank** | **Bootstrap mean±SD** | **Min rank** | **Max rank** | **Stable?** |
| --- | --- | --- | --- | --- | --- | --- |
| P9 | 0.9645 | 1 | 1±0 | 1 | 1 | Yes |
| P8 | 0.7263 | 2 | 2±0.03 | 2 | 3 | Yes |
| P1 | 0.5852 | 3 | 3.16±0.36 | 3 | 4 | Yes |
| P3 | 0.5031 | 4 | 3.84±0.37 | 2 | 4 | Yes |
| P2 | 0.2638 | 5 | 5.06±0.23 | 5 | 6 | Yes |
| P4 | 0.1120 | 6 | 5.94±0.23 | 5 | 6 | Yes |
| P5 | -0.3711 | 7 | 7±0 | 7 | 7 | Yes |
| P10 | -0.5909 | 8 | 8.29±0.45 | 8 | 9 | Yes |
| P6 | -0.6148 | 9 | 8.71±0.45 | 8 | 9 | Yes |
| P7 | -0.8429 | 10 | 10±0 | 10 | 10 | Yes |

*Bootstrap: 1,000 resampling iterations with replacement. Weight perturbation analysis (±20%) confirmed identical ranking stability. All analyses performed on real batch-level data from the laboratory notebook.*
